# Supplementary material for: Genomic Instability in Cerebrospinal Fluid Cell-Free DNA Predicts Poor Prognosis in Solid Tumor Patients with Meningeal Metastasis
Source: Cancers (Basel). 2022 Oct 14;14(20):5028. doi: 10.3390/cancers14205028 (PMC9600191; doi:10.3390/cancers14205028)
Supplement: Supplementary file 1 [file cancers-14-05028-s001.zip › cancers-1913915-supplementary.pdf]

cancers MDPI

Supplementary Materials: Genomic Instability in Cerebrospinal Fluid Cell-Free DNA Predicts Poor Prognosis in Solid Tumor Patients with Meningeal Metastasis

Peng Wang, Qiaoling Zhang, Lei Han, Yanan Cheng, Zengfeng Sun, Qiang Yin, Zhen Zhang, Jinpu Yu

Table S1. Distribution of variation in the top 35 genes for different genomic status.

Characteristic	GI, N = 37 [1]	GS, N = 19 [1]	p-Value [2]
TP53			<0.001
variant	29 (78%)	6 (32%)	
wildtype	8 (22%)	13 (68%)	
CDKN2A			0.077
variant	10 (27%)	1 (5.3%)	
wildtype	27 (73%)	18 (95%)	
APC			0.082
variant	7 (19%)	0 (0%)	
wildtype	30 (81%)	19 (100%)	
RICTOR			0.11
variant	18 (49%)	5 (26%)	
wildtype	19 (51%)	14 (74%)	
KMT2C			0.14
variant	9 (24%)	1 (5.3%)	
wildtype	28 (76%)	18 (95%)	
ERBB2			0.15
variant	8 (22%)	1 (5.3%)	
wildtype	29 (78%)	18 (95%)	
RB1			0.15
variant	8 (22%)	1 (5.3%)	
wildtype	29 (78%)	18 (95%)	
EGFR			0.2
variant	28 (76%)	11 (58%)	
wildtype	9 (24%)	8 (42%)	
SMAD4			0.2
variant	5 (14%)	0 (0%)	
wildtype	32 (86%)	19 (100%)	
FGFR4			0.3
variant	4 (11%)	0 (0%)	
wildtype	33 (89%)	19 (100%)	
SETD2			0.3
variant	4 (11%)	0 (0%)	
wildtype	33 (89%)	19 (100%)	
STAG2			0.3
variant	2 (5.4%)	3 (16%)	
wildtype	35 (95%)	16 (84%)	
STK11			0.3
variant	4 (11%)	0 (0%)	
wildtype	33 (89%)	19 (100%)	

RBM10			0.4
variant	6 (16%)	1 (5.3%)	
wildtype	31 (84%)	18 (95%)	
CDH1			0.4
variant	3 (8.1%)	3 (16%)	
wildtype	34 (92%)	16 (84%)	
KMT2D			0.4
variant	3 (8.1%)	3 (16%)	
wildtype	34 (92%)	16 (84%)	
MYC			0.4
variant	6 (16%)	1 (5.3%)	
wildtype	31 (84%)	18 (95%)	
DNMT3A			0.6
variant	2 (5.4%)	2 (11%)	
wildtype	35 (95%)	17 (89%)	
MTOR			0.6
variant	2 (5.4%)	2 (11%)	
wildtype	35 (95%)	17 (89%)	
NF1			0.6
variant	2 (5.4%)	2 (11%)	
wildtype	35 (95%)	17 (89%)	
TERT			0.6
variant	2 (5.4%)	2 (11%)	
wildtype	35 (95%)	17 (89%)	
BRCA2			0.7
variant	5 (14%)	1 (5.3%)	
wildtype	32 (86%)	18 (95%)	
CDK4			0.7
variant	6 (16%)	2 (11%)	
wildtype	31 (84%)	17 (89%)	
GNAS			0.7
variant	4 (11%)	1 (5.3%)	
wildtype	33 (89%)	18 (95%)	
KRAS			0.7
variant	5 (14%)	1 (5.3%)	
wildtype	32 (86%)	18 (95%)	
NOTCH1			0.7
variant	4 (11%)	1 (5.3%)	
wildtype	33 (89%)	18 (95%)	
PTEN			0.7
variant	7 (19%)	2 (11%)	
wildtype	30 (81%)	17 (89%)	
PIK3CA			0.8
variant	10 (27%)	4 (21%)	
wildtype	27 (73%)	15 (79%)	
BAP1			>0.9
variant	3 (8.1%)	2 (11%)	
wildtype	34 (92%)	17 (89%)	
BRCA1			>0.9
variant	3 (8.1%)	1 (5.3%)	
wildtype	34 (92%)	18 (95%)	
CDK12			>0.9
variant	3 (8.1%)	1 (5.3%)	
wildtype	34 (92%)	18 (95%)	

FGFR1			>0.9
variant	3 (8.1%)	2 (11%)	
wildtype	34 (92%)	17 (89%)	
IRS2			>0.9
variant	3 (8.1%)	1 (5.3%)	
wildtype	34 (92%)	18 (95%)	
MDM2			>0.9
variant	3 (8.1%)	1 (5.3%)	
wildtype	34 (92%)	18 (95%)	
NOTCH2			>0.9
variant	3 (8.1%)	1 (5.3%)	
wildtype	34 (92%)	18 (95%)	

[1] n (%)

[2] Pearson's Chi-squared test; Fisher's exact test

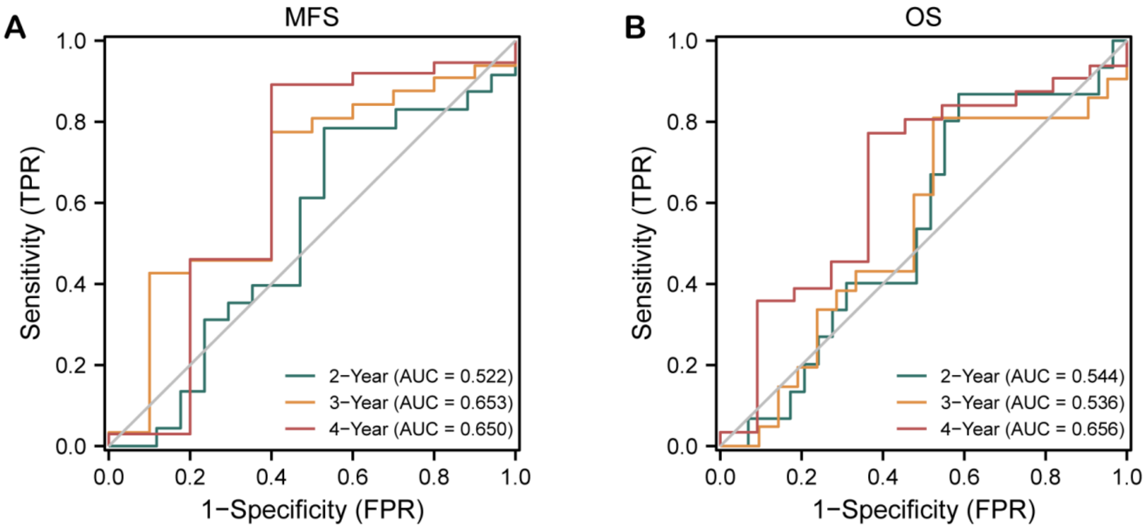

Figure S1. Receiver operating characteristic (ROC) curves for MFS and OS.

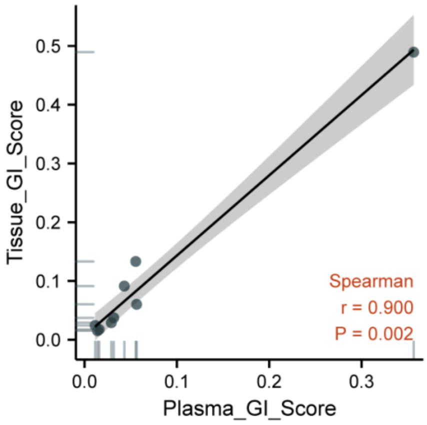

Figure S2. Correlation analysis of GI_Scores between 9 paired plasma cfDNA and primary tumor tissues.
